# Are human embryos Kantian persons?: Kantian considerations in favor of embryonic stem cell research

**DOI:** 10.1186/1747-5341-3-4

**Published:** 2008-01-31

**Authors:** Bertha Alvarez Manninen

**Affiliations:** 1 Department of Integrative Studies, New College of Interdisciplinary Arts and Sciences, Arizona State University at the West Campus, Phoenix, AZ USA

## Abstract

One argument used by detractors of human embryonic stem cell research (hESCR) invokes Kant's formula of humanity, which proscribes treating persons solely as a means to an end, rather than as ends in themselves. According to Fuat S. Oduncu, for example, adhering to this imperative entails that human embryos should not be disaggregated to obtain pluripotent stem cells for hESCR. Given that human embryos are Kantian persons from the time of their conception, killing them to obtain their cells for research fails to treat them as ends in themselves.

This argument assumes two points that are rather contentious given a Kantian framework. First, the argument assumes that when Kant maintains that humanity must be treated as an end in itself, he means to argue that all members of the species *Homo sapiens *must be treated as ends in themselves; that is, that Kant regards personhood as co-extensive with belonging to the species *Homo sapiens*. Second, the argument assumes that the event of conception is causally responsible for the genesis of a Kantian person and that, therefore, an embryo is a Kantian person from the time of its conception.

In this paper, I will present challenges against these two assumptions by engaging in an exegetical study of some of Kant's works. First, I will illustrate that Kant did not use the term "humanity" to denote a biological species, but rather the capacity to set ends according to reason. Second, I will illustrate that it is difficult given a Kantian framework to denote conception (indeed any biological event) as causally responsible for the creation of a person. Kant ascribed to a dualistic view of human agency, and personhood, according to him, was derived from the *supersensible *capacity for reason. To argue that a Kantian person is generated due to the event of conception ignores Kant's insistence in various aspects of his work that it is not possible to understand the generation of a person *qua *a physical operation. Finally, I will end the paper by drawing from Allen Wood's work in Kantian philosophy in order to generate an argument in favor of hESCR.

## Introduction

One argument constantly used by detractors of human embryonic stem cell research invokes Immanuel Kant's formula of humanity, the second principal formulation of the categorical imperative. In the *Groundwork of the Metaphysics of Morals *(*Groundwork*, from hereonin), Kant states the second principle as follows:

Now I say that the human being and in general every rational being exists as an end it itself, *not merely as a means *to be used by this or that will at its discretion; instead he must in all his actions, whether directed to himself or also to other rational beings, always be regarded *at the same time as an end*... The practical imperative will therefore be the following: *So act that you use humanity, whether in your own person or in the person of any other, always at the same time as an end, never merely as a means *[1].

In "The Stem Cell Slide: Be Alert to the Beginnings of Evil," Michael Novak uses the formula of humanity as the theoretical ground for rejecting the use of human embryos for stem cell research. Novak writes:

You must never use a human being as a means for even the noblest of ends. To use stem cells obtained by killing living human beings in their embryonic stage is still using them as a means. It is not enough to say that the wicked deed has been done – that the embryos have already been killed. The purpose of that killing was to obtain the stem cells. One ought not to implicate oneself in that process, not even for the noblest and most beautiful ends [2].

Of course, Novak cannot literally mean what he says: that we must never use human beings as means to even noble ends. To follow Novak's suggestion would be utterly impossible, given that human beings use each other as means to several ends all the time. Kant does not proscribe treating persons as a means to an end, rather he argues against persons treating each other *solely *as a means to an end; in a manner that completely dehumanizes them. It is certainly permissible for persons to treat each other as means *so long as they are, simultaneously, treating each other as ends in themselves*.^a ^Therefore, I will assume that what Novak means here is that destroying embryos for stem cell research completely dehumanizes them and treats them *solely *as a means to an end.

In his August 9, 2001 speech to the nation regarding the federal funding (or lack thereof) of human embryonic stem cell research, George W. Bush alluded to the formula of humanity as well when he stated that " [e]ven the most noble ends do not justify any means... the fact that a living being is going to die does not justify experimenting on it or exploiting it as a natural resource"[3]. Similar arguments abound elsewhere in the stem cell literature. Physician, philosopher, and theologian Fuat S. Oduncu argues that:

The human embryo is looked upon as a human being from the moment of its conception and thus attributed the fundamental principle of human dignity that guarantees the right to life of the embryo. According to Kant, human dignity forbids and even condemns instrumentalization and reduction of a human being to a mere means and object. Human beings are persons and as such they are ends in themselves... [t]he mere membership of humanity creates and preserves the fundamental value of human dignity until death... [s]ince the living human embryo is the very first concrete and individual agent in human development, it must be regarded as the carrier of implicit and unconditional values [4].

Jens G. Reich writes that the formula of humanity "excludes categorically any instrumentalization of a human being for means other than its own existence, thus prohibiting procreation of a human embryo solely for scientific or medical purposes" [5]. Paul R. Boehlke invokes Kant's imperative as well in order to also argue that each human embryo should be treated as an end in itself and never as a means only [6].

Each of the abovementioned authors assume a very crucial, yet highly contentious, premise: that when Kant argues that humanity must be treated as an end in itself, that he means to denote a biological category, i.e., that that all *Homo sapiens *must be treated as ends in themselves. Moreover, the arguments above, most explicitly Oduncu's argument, assume that it is at conception (the time when a sperm fully combines with the ovum to form a new and distinct genetic code) that a new Kantian person comes into existence; that the newly conceived human zygote (particularly, at the blastocyst stage of development, about five days after fertilization) qualifies as the type of being to whom the second categorical imperative applies.

The purpose of this paper is three-fold. First, I will illustrate how, in the second categorical imperative, Kant does not use the term "humanity" to denote a certain biological category, but rather a certain human capacity, i.e, the capacity for reason. Therefore, what must be treated as an end in itself is not necessarily a human being *simplicter*, but rather the capacity for reason *within *a human being. Second, I will expand on an argument briefly mentioned by Mark Sagoff in his article "Extracorporeal Embryos and Three Conceptions of the Human": that it would be difficult to regard conception, indeed *any *biological event, as the exact time when a Kantian person comes into existence. My purpose in the first two sections of the paper is largely to illustrate that the ascription of Kantian personhood to human embryos from the time of their conception is more problematic than is assumed by the abovementioned authors, and thus that they, or anyone that happens to agree with their contention, need to provide a better argument to establish this crucial premise. It is important to note that I am *not *arguing that human embryos are definitely not Kantian persons. Rather my goal is much more modest; my main concern is to illustrate the difficulties that come with making the claim that the act of conception realizes a Kantian person in the world. By doing so, I will, in effect, be throwing the ball back into the court of philosophers like Oduncu and Novak; they would need to at least address the difficulties I pose in this paper before contending that Kantian personhood applies to embryos from the time of their conception. Lastly, I will appeal largely to Allen Wood's work on Kantian philosophy in order to sketch a Kantian argument in favor of embryonic stem cell research.

## The Kantian definition of 'humanity' and 'person'

Let us look closer at the exact wording of the formula of humanity: "act that you use humanity, whether in your own person or in the person of any other, always at the same time as an end, never merely as a means."

Kant defines a "person" in the *Groundwork *not as a member of the species *Homo sapiens, *but rather as a rational being whose "nature already marks [him] as an end in itself, that is, as something that may not be used merely as a means and hence so far limits all choice (and is an object of respect)" [7]. In the *Metaphysics of Morals *(*Metaphysics*, from hereonin), Kant describes a person as "a subject whose actions can be *imputed *to him. *Moral *personality is therefore nothing other than the freedom of a rational being under moral laws" [8]. From these lines alone, it seems that Kant considered the term "person" to be applicable to beings with certain capacities, rather than to beings who are members of a certain species. Indeed, it is notable that, despite his attitude toward nonhuman animals (which will be explored below), Kant was not opposed to the idea that there could be nonhuman persons, i.e., nonhumans in possession of a rational nature [9].

A closer inspection of Kant's use of the term "humanity" also reveals that he did not use the term in a descriptive sense to pick out all and only *Homo sapiens*. Rather, the term "humanity" denotes a certain capacity or predisposition in persons.^b ^In *Religion Within the Boundaries of Mere Reason*, Kant describes three elements of human nature: animality, humanity, and personality [10]. "Animality" is part of human nature simply in virtue of our being live human *animals*, "it is the basis for our fundamental instinctual drives aiming at self-preservation (the drives for food, bodily well-being, and so on)" [11]. "Personality" is the capacity or predisposition that is the source of our dignity as rational creatures; it denotes our ability to respect the moral law and perform our duty solely because of duty's sake (i.e., it denotes our ability to possess a good will). Kant refers to personality as "the susceptibility to respect for the moral law as *in itself a sufficient incentive of the power of choice*" [12]. As Allen Wood notes: "Kant identifies personality with autonomy, in the sense of the ability to give oneself the moral law through reason, which is the ground of dignity" [13].

According to Kant, "humanity" is also a predisposition or a capacity, rather than a species denotation, and it refers to the rational faculties of persons, the ability that persons possess to follow self-imposed ends. Unlike the capacity for personality, which contains as part of its definition respect for the moral law, the capacity for humanity is the capacity for reason *proper*, without any explicit reference to morality.^c ^Although the predisposition for personality is the source of the dignity of persons, the reason why it is *humanity *that must be treated as an end in itself is because Kant emphasizes that it is rational nature *simplicter*, not rational nature being exercised in a certain manner, that must be respected. In the *Metaphysics*, Kant writes:

The capacity to set oneself an end – any end whatsoever – is what characterizes humanity (as distinguished from animality). Hence, there is also bound up with the end of humanity in our own person the rational will, and so the duty, to make ourselves worthy of humanity by culture in general, by procuring or promoting the *capacity *to realize all sorts of possible ends, so far as this is to be found in a human being himself [14].

That is, " [p]reserving and respecting rational nature means preserving and respecting it in all its functions, not merely in its moral function of giving and obeying moral laws. Furthering rational nature requires furthering all the (morally permissible) ends it sets, not merely in its moral function of giving and obeying rational laws" [15]. For example, in the *Metaphysics *Kant argues that all persons have an imperfect duty to cultivate one's talents. Kant writes:

A human being has a duty to himself to cultivate (*cultura*) his natural powers (powers of spirit, mind, and body), as means to all sorts of possible ends. – He owes it to himself (as a rational being) not to leave idle and, as it were, rusting away the natural predispositions and capacities that his reason can someday use [16].

Notice that the duty to the self is to cultivate one's rational faculties *simplicter*; Kant makes no mention of cultivating *only *one's moral prowess. When it comes to our duties toward others, Kant also maintains that each rational being must make the rational (morally permissible) ends of other persons " [their] own end as well" [17]; the promotion of the ends of other rational creatures is also my duty. No mention is made that I must respect or promote other people's ends *only *when they are related to following the moral law; I must respect the rational or autonomous decision that another person makes because in doing so, he is exercising his humanity.

Once it is understood what Kant means by the terms "person" and "humanity," the formula of humanity can be re-phrased as follows: "act that you use *the capacity or predisposition for reason*, whether in your own person or in the person of any other, always at the same time as an end, never merely as a means." The object of respect in the formula of humanity, therefore, is not a certain biological species, but rather a certain capacity. Kant reiterates this imperative several times throughout his writings. In the *Metaphysics*, for example, Kant argues that all human beings are required to "acknowledge, in a practical way, the dignity of humanity in every other human being. Hence, there rests on him a duty regarding the respect that must be shown to every other human being" [18]. Notice what Kant does *not *say. He does *not *just say that we are to respect the dignity of every other human being. Rather, he says that we are to respect the dignity of humanity (the capacity for reason) *in *every human being and that *therefore *we must respect a human being *in virtue *of the fact that he possesses this capacity. If Kant wanted to argue that we must respect every single member of the species *Homo sapiens *as an end in himself, surely the former method of wording the imperative would have sufficed. Yet Kant takes pains to always remind us that it is the humanity *within *human beings or *within *persons that must be respected. That is, what must be respected is the capacity for reason; the capacity to set ends, follow those ends, and be an autonomous individual.

Therefore, from the wording of the imperative alone, it is not true, *contra *Oduncu, that Kant "condemns the instrumentalization and reduction of a human being [*qua Homo sapiens*] to a mere means and object" and it is also not true, from the wording of this imperative alone, that " [t]he mere membership of humanity creates and preserves the fundamental value of human dignity until death." The term "humanity" is not meant to denote a membership in any group at all, rather it denotes a certain capacity possessed by all persons, i.e., accountable beings, to reason and set ends for themselves. One need not be human, in the biological sense, to be a person, according to Kant. Thus, the term "person" or "humanity" is not necessarily tied into the concept of *Homo sapiens *in Kantian moral philosophy. From the second principle formulation of the categorical imperative alone, it cannot be concluded that mere species membership is sufficient grounds for Kantian personhood, and thus sufficient grounds for dignity or respect in the sense of not being treated as a mere means. Therefore, from this alone, we cannot conclude that human blastocysts are included in Kant's sense of the term "person" and thus that they must be treated as ends in themselves.

Nevertheless, the question of whether an embryo or a fetus may still be counted as a person who possesses the predisposition or capacity for reason is worth pursuing in light of other aspects of Kant's writing. Kant did consider children persons, even if their humanity, their capacity for reason, and their personality, their capacity to follow the moral law, are not quite developed. Indeed, Kant refers to small children as persons who have an innate right to their parent's care [19] even though small children are not the type of beings "whose actions can be imputed to [them]." The correct question that philosophers like Novak and Oduncu need to ask is whether there are Kantian reasons for thinking that a human embryo or fetus, from conception, has the capacity for reason as part of its nature, and therefore that it possesses humanity, even if it is not a capacity that is currently being exercised.^d ^If so, perhaps a case can be made that human embryos ought to be treated as ends in themselves, not because of their species membership, but instead because they too possess the capacity that Kant so adamantly champions as an object of respect.

## The significance of conception and Kant's Third Antinomy

Individuals who argue that blastocyst-stage embryos possess value akin to any other human person usually argue that this value is acquired at conception. Given that we are approaching this from a Kantian standpoint, what seems to be assumed by these individuals is that the moment of conception (although this is a misnomer, since conception does not take place in a single moment) realizes a new being in the world that possesses the capacity for reason as part of its nature and is, therefore, a proper subject of Kantian moral concern.

Let us take a closer look at Oduncu's argument:

The human embryo is looked upon as a human being from the moment of its conception and thus attributed the fundamental principle of human dignity that guarantees the right to life of the embryo. According to Kant, human dignity forbids and even condemns instrumentalization and reduction of a human being to a mere means and object. Human beings are persons and as such they are ends in themselves... [t]he mere membership of humanity creates and preserves the fundamental value of human dignity until death... the author of these lines proposes to relinquish the notorious concept of "the person" in order to promote the clarity and quality of the bioethical debate on the research with human embryos. Since the concept of the "human being" is more fundamental, and even precedes the concept of "the person," the bioethical debate should be conducted on the grounds of a biological and anthropological concept of the human being. The concept of the human being and the inherent value of human dignity related to it may find a wider acceptance, as it refers to the biological species of the Homo sapiens sapiens [20].

Here are the following claims that, it seems to me, can be derived from the above passage. First, Oduncu argues that the event of conception is responsible for generating a human being in the world with inherent human dignity. Given that Oduncu is appealing to Kantian philosophy, I will assume that his concept of human dignity is akin to Kant's. Therefore, it seems that what Oduncu means to say is that the event of conception is causally responsible for generating a human being in the world that possesses the capacity for reason; conception is causally responsible for the genesis of the Kantian person. Indeed, if he does not think this, he does not give an alternative viewpoint concerning the genesis of the Kantian person. Since he does not mention any other biological event other than conception, it seems safe to assume that he means to say that conception is responsible for generating a Kantian person. Second, it seems clear that Oduncu wishes to equate the concept of a Kantian person with the species *Homo sapiens*. Indeed, he argues that the ethical debate concerning the moral status of the human embryo "should be conducted on the grounds of a biological and anthropological concept of a human being." I am not sure what Oduncu means by "anthropological" here (or whether this is in harmony with how Kant approaches anthropological studies), but it does seem rather clear that he wishes to argue that we should understand the genesis of a human being, *and therefore a Kantian person*, as occurring due to the event of conception.

Oduncu wishes to understand the origins of a Kantian person via a biological event. The problem, as I will illustrate below, is that the capacity for reason, according to Kant, is a supersensible capacity, given that the possession of transcendental freedom is a necessary precondition for possessing this capacity. Therefore, the argument that must be made by philosophers like Oduncu, in order to stay true to the Kantian conception of personhood, is that conception is the moment that realizes, or brings into existence, a being who possesses the supersensible capacity for reason (albeit, perhaps, in a latent form). Is there any textual evidence in Kant's philosophy that lends credence to such a view? Some philosophers have certainly argued as much, and those arguments are rather impressive and formidable ones.^e ^Mark Sagoff, however, maintains quite the opposite. He writes:

It is impossible to attribute moral status to an [extracorporeal human embryo] on grounds of its physical characteristics alone – even when its potential is considered – because there is no point in the process of ontogeny at which a scientific finding can be made, as it were, that a glob of protoplasm is now sufficiently endowed with moral freedom that it has become a responsible agent or sufficiently endowed with cultural, aesthetic, and ethical capacities that it has become a human being [21].

Given that a human embryo is obviously a human being in the biological sense, I assume that what Sagoff means here is that mere biological humanity cannot tell us anything about *moral *humanity; about what it means to be a person, or when a human being becomes a person. In other words, although biological humanity is an observable, physical, trait, Kantian personhood (a being endowed with "moral freedom that... has become a responsible agent") is not. In order to support this claim, Sagoff appeals to Kant's metaphysics of human freedom as discussed in the Third Antinomy in his *Critique of Pure Reason *(first *Critique*, from hereonin):

Kant, of course, struggled in the Third Antinomy and elsewhere with the disconnection between the empirical self (the subject of scientific or biological research) and the intelligible self (the subject of agency, freedom, judgment, *and thus moral status*). Kant understood that from a biological or scientific perspective, all natural activities (whether of humans or non-humans, adults, or embryos) have to be explained in terms of the deterministic freedom-excluding framework of the physical and chemical causality. Kant believed that to show we can act as moral and cultural beings – to show that we are not all bound by natural law as are stones – is already to accomplish a lot and much more than can be inferred from any biological or physiological inquiry [22].

Here, Sagoff argues that the aspect of a human being that possesses moral worth in the Kantian framework, the rational agent or the "intelligible self," is not reducible to the natural or "empirical" aspect of the human being. In what follows, I will offer support of Sagoff's claim by engaging in an exegesis of Kant's first *Critique*, along with other supporting literature.

As discussed in the previous section, although Kant argues that it is the predisposition for personality that is the source of human dignity, it is the predisposition for humanity, the ability to make rational decisions, that must be treated with respect and as an end in itself. According to Kant, with the ability to make rational choices comes another very important feature: freedom. As Wood notes, the possession of the predisposition for humanity "presupposes a kind of freedom, namely the ability to resist the immediate coercion of desires and impulses" [23]. Therefore, the capacity for humanity entails the possession of freedom, and, as Sagoff argues, there is much reason to believe that Kant would have resisted identifying conception, indeed *any *biological milestone, as the precise time when a being endowed with freedom is realized in the world.

In the first *Critique*, Kant discusses the problem of free will versus physical determinism as the Third Antinomy of Pure Reason. Kant acknowledges that there is a tension between humans possessing freedom and the fact that humans are subject to physical causal laws; if all human action is determined by the latter, then the former does indeed appear to be impossible. Kant expresses the problem in the following way:

Suppose there were a freedom in the transcendental sense, as a special kind of causality in accordance with which the occurrences of the world could follow, namely a faculty of absolutely beginning a state, and hence also a series of consequences; then not only will a series begin absolutely through this spontaneity, but the determination of this spontaneity itself to produce the series, i.e., its causality, will begin absolutely, so that nothing precedes it through which this occurring action is determined in accordance with constant laws. Every beginning action, however, presupposes a state of the not yet acting cause, and a dynamically first beginning of action presupposes a state that has no causal connections at all with the cause of the previous one, i.e., in no way follows from it. Thus transcendental freedom is contrary to the causal law ... [24]

Freedom, Kant maintains, cannot be tied with any empirical, natural, phenomenon, for the latter necessarily entails a determinate chain of causality, while freedom presupposes no causal determinism. Kant is, in essence, characterizing freedom in a rather libertarian sense, and he calls it "transcendental freedom." As Henry Allison notes, the exercise of freedom, according to Kant, must "involve an element of spontaneity" [25]; a true exercise of freedom cannot be causally determined in any sense.

According to Kant, even though all empirical evidence establishes otherwise, transcendental freedom must exist, for the fact that persons acknowledge the moral law provides indirect evidence that this is the case. In the *Critique of Practical Reason *(second *Critique*, from hereonin), Kant argues that:

... the moral principle, conversely itself serves as the principle of deduction of an inscrutable faculty which no experience could prove but which speculative reason had to assume as at least possible... namely, the faculty of freedom, of which the moral law, which itself has no need for justifying grounds, proves not only the possibility but the reality in beings who cognize this law as binding upon them [26].

Our empirical exposure to the world cannot justify assuming the faculty of freedom because the exercise of (the Kantian notion of transcendental) freedom must be liberated from causal laws, and all experience tells us that nothing in the empirical world is liberated from causal laws. Therefore, insofar as human beings are subject to the empirical world, and insofar as we are natural creatures, the possession of freedom is impossible. Nevertheless, freedom must be assumed to exist if human beings recognize the moral law as binding upon them, since the moral law requires that we follow it independently of what our desires and inclinations demand. Allison affirms this Kantian connection between freedom and morality in what he calls the "Reciprocity Thesis... the claim that freedom and the moral law reciprocally imply one another" [27].

Kant argues that such a tension between freedom and natural causality can only be resolved once we draw the distinction between the natural/phenomenal and the intelligible/noumenal; between what Kant calls the "phenomenal self" (the empirical self) and the "noumenal self" (the purely intelligible self). Here, Kant argues that human beings possess a dualistic nature;^f ^there is an aspect to human nature that is purely intelligible and independent from the physical/empirical world. The part of the human being that is a member of the empirical world, the phenomenal self, is subject to the causal laws of nature. However, the intelligible self, the part of a human being that exercises freedom, cannot be found in the empirical self, but rather in the noumenal self, the part of the human being that is independent from the empirical world and therefore the laws of causality. It is because humans possess a noumenal side, according to Kant, that we are able to follow the moral law despite our inclinations and desires. Human beings are not completely ruled by those inclinations and desires because our reason allows us to be free of them if we so choose, and we can follow the moral law simply because we recognize it as binding on all rational persons. Kant further supports this dualistic view of human agency in the *Anthropology from a Pragmatic Point of View*, where he writes that human beings have two types of character: " [t]he first is the distinguishing mark of the human being as a sensible or natural being; the second is the distinguishing mark of the human being as a rational being endowed with freedom" [28].

Recalling that a person, according to Kant, is a being whose actions can be imputed onto him, a person is a being who possesses this noumenal, intelligible, aspect to his agency, a person is a "rational being endowed with freedom." Allison puts it well when he writes:

From this perspective we can impute the actions to an agent and claim that they ought or ought not to have been performed. This, Kant suggests, is because in viewing actions in this manner we are considering them in relation to "something intelligible," which stands outside of the temporal order of the phenomenal world. This is, of course, the agent's practical spontaneity, his capacity to act on the basis of reason, which is assigned to his intelligible character [29].

A person, according to Kant, is a being that possesses this transcendental, intelligible, aspect to his character; an aspect to his character that cannot be reduced to the phenomenal/empirical world. In making this claim, Kant has effectively argued that a being's personhood is not reducible to any of his physical aspects, but, rather, that personhood belongs to the transcendental, intelligible, aspect of a human being. Hence, Kant has effectively severed the attainment of personhood from any physical process or occurrence. A being's possession of personhood, rather, can only be understood by appealing to his transcendental, noumenal, self.

According to Kant, therefore, freedom and autonomy, and with it the capacity and predisposition for humanity and personality (given that the latter two contain the former two as necessary components), are not part of our physical, empirical, phenomenal, self; rather they are part of our transcendental, intelligible, noumenal, self. As Allison puts it, it is only in the idea of the transcendental aspects of humanity, rather than the empirical aspects of humanity, that Kant "provides a model for conceiving of human choice or agency... Kant's point is that the conceivability of practical freedom necessarily involves a reference to the transcendental Idea" [30].

Within Kant's corpus, there is a distinction between transcendental and practical freedom; many Kantian scholars contend that Kant's ethical writings are more concerned with the latter rather than the former, and thus that it is the possession of the latter that is necessary in order for an individual to be a moral subject from a Kantian perspective. Kant defines practical freedom in the *Groundwork *as the "property of such causality that it can be efficient independently of alien causes determining it, just as natural necessity is the property of the causality of all nonrational beings to be determined to activity by the influences of alien causes... [freedom] is not a property of the will in accordance with natural laws" [31]. Even in his practical philosophy, Kant considers human freedom (and human reason) as immune from the influence of inclinations or empirical laws of causation. Indeed, in the *Groundwork *Kant maintains that humans possess dignity in virtue of their ability to be "free with respect to all laws of nature, obeying only those which he himself gives and in accordance with which his maxims can belong to a giving of universal law (to which at the same time he subjects himself)" [32]. Although practical freedom seems to be the main concern in Kant's moral philosophy, the aforementioned discussion concerning transcendental freedom is far from moot. As Allison's quote above illustrates, it is not possible to understand the nature of practical freedom, according to Kant, without a reference to transcendental freedom. Indeed, in the first *Critique*, Kant explicitly maintains that "it is this transcendental idea of freedom on which the practical concept of freedom is grounded... the abolition of transcendental freedom would also simultaneously eliminate all practical freedom" [33]. Therefore, the existence of practical freedom is at least conceptually dependent on transcendental freedom, and, like transcendental freedom, practical freedom involves independence from empirical or natural causality: "freedom in the practical sense is the independence of the power of choice from necessitation by impulses of sensibility" [34] All beings who possess freedom in the practical sense also possess an intelligible aspect of the self, the part of the self that is not reducible to the empirical world.

Because Kant argues that human freedom and autonomy belong to some transcendental realm and not to any empirical or physical realm, Sagoff seems to be accurate in his claim that it is difficult to argue, from a Kantian perspective, that it is because of conception, an empirical and physical event, that the transcendental, intelligible, noumenal, part of the self, i.e., the *person*, is also generated. Essentially, arguing that the empirical event of conception is responsible for the realization of a new creature in the world who possesses the supersensible capacity for reason seems to ignore Kant's dualistic conception of human agency. At the very least, this renders the transcendental capacity for freedom and autonomy intimately connected with the empirical world in a manner that may be too close for comfort from a Kantian perspective.

One very important line in the *Metaphysics *that supports Sagoff's, and my own, reading of Kant is when he argues that:

... the offspring is a person, *and it is impossible to form a concept of the production of a being endowed with freedom through a physical operation *[35].

Conception is a purely physical operation, and here Kant explicitly states that it is impossible to conclude that a being endowed with freedom comes into existence from a purely physical operation. Indeed, Kant is so committed to the idea that the genesis of a free being cannot be accounted for by a purely physical occurrence that he even argues that:

No concept can be formed of how it is possible for God *to create *free beings, for it seems as if all their future actions would have to be predetermined by that first act, included in the chain of natural necessity and therefore not free. But that such beings (we human beings) are still free the categorical imperative proves for morally practical purposes... [a]ll that one can require of reason here would be merely to prove that there is no contradiction in the concept of a *creation of free beings *[36].

According to Kant, as is evidenced in the first *Critique*, the tension between the existence of beings that are simultaneously free and also subject to the deterministic laws of nature vanishes only with the distinction between the phenomenal/empirical and the noumenal/intelligible. To denote conception as the event that is causally responsible for the existence of a being who is endowed with the predisposition for humanity *qua *the capacity to make free and rational choices seems to contradict Kant's above statement that we cannot understand the production of a free being from a purely physical operation, and it is certainly at odds with his conclusion in the Third Antinomy that we cannot account for the existence of transcendental freedom by appealing to the phenomenal world.

The following claims should make clearer how an argument like Sagoff's can be developed and contrasted to Oduncu's claims about the personhood of the human blastocyst from a Kantian perspective.

1. A Kantian person is a being who is necessarily endowed with the capacity for reason.

2. All beings who possess the capacity for reason do so in virtue of their intelligible/noumenal self; the part of the self that is transcendentally free.

3. Therefore, all Kantian persons possess the capacity for reason in virtue of their intelligible/noumenal self; the part of the self that is transcendentally free.

4. It is impossible to account for the existence of the noumenal self by appealing to an empirical event as casually responsible for its existence (because if we did so, the noumenal self would be subject to the causal laws of nature and would therefore necessarily cease to be part of the noumenal world).

5. Therefore, from a Kantian perspective, it is impossible to account for the existence of the person by appealing to an empirical event as casually responsible for its existence.

6. Conception, being an empirical event, cannot be appealed to as being causally responsible for the generation of the Kantian person because this would then render the Kantian person subject to the causal laws of nature. This would violate the essential characteristic of a Kantian person: an intelligible (rather than empirical) being who is not subject to causal laws and is, therefore, a rational agent endowed with freedom.

7. Therefore, *contra *Oduncu, it is not possible to argue, from a Kantian perspective, that conception is causally responsible for the creation of a being endowed with the transcendental, supersensible, capacity for reason.

With all this, Sagoff's argument becomes clearer, and I believe that it does pose a formidable obstacle for philosophers such as Oduncu and Novak who wish to ascribe such moral import to the event of conception. It is certainly at conception that the empirical or material aspect of a human being begins to exist. However, Oduncu and Novak seem to infer that the intelligible aspect of the self is also generated because of conception. Yet, if conception is causally responsible for the creation of the Kantian person, the intelligible self, then this renders the creation of the intelligible self, along with the creation of the empirical self, a physical phenomenon. This means that the intelligible self would also be subjected to the deterministic laws of nature, would not possess transcendental freedom, and therefore would cease to be an intelligible self. Hence, we simply cannot look towards any empirical occurrence in order to account for the existence of the intelligible, noumenal self, the Kantian person. To argue that conception is causally responsible for the generation of the noumenal self would be to enslave the noumenal self to the physical chain of causality that Kant argues it is necessary that it exist independently of in order to account for the possibly of freedom, autonomy, and rational and moral agency. In short, while Oduncu wants to conceive of Kantian personhood and human dignity as empirical and biological, Kant's conception of personhood and human dignity, being so dependent on transcendental freedom, is supersensible in nature.

It is true that Kant does call the offspring a person, and he argues that we should regard the "act of procreation as one by which we have brought a person into the world without his consent and on our own initiative, for which deed the parents incur an obligation to make the child content with his condition so far as they can" [37]. Kant also argues that parents possess this duty "from procreation" and that "children, as persons, have by their procreation an original innate (not acquired) right to the care of their parents [38]. Although Kant maintains that the act of procreation results in bringing a person into the world, this is *not *equivalent to arguing that a person exists as soon as procreation is completed (for example, I can say that "The act of planting an acorn is one by which we have brought an oak tree into the world" but this is not the same as arguing that the oak tree exists as soon as the acorn is planted). Wood makes this distinction as well in his book *Kantian Ethics*:

Of course it is one thing to say that parents should be thought of as bringing a person into being, and even that they have duties of care to their offspring from conception. It is quite a different thing to say that the offspring is a *person *from conception onward. The first two things Kant does appear to say; the third is something he never quite says [39].

The act of procreation certainly results in the beginning of a unique genetic code that will constitute a person, but it need not mean that procreation is the precise moment when the person is created. It is also possible to account for the parental duty to one's child from procreation because it is at procreation when the empirical, phenomenal, self begins to exist, and the transcendental aspect of the child, the being endowed with freedom, cannot exist without the empirical aspect of the child existing. Thus, in order to ensure the genesis of the child *qua *person (as the being endowed with transcendental and practical freedom, and therefore humanity and personality), we must care for the child *qua *empirical being, which begins to exist at procreation. But, again, this is distinct from arguing that it is precisely at procreation when the transcendental aspect of the human being, the part of the human being that possesses freedom and rational agency, and is therefore a person, begins to exist.

Finally, I would like to make clear what I am *not *arguing. I am not arguing that it is the case that Kantian moral philosophy positively prohibits the ascription of personhood to embryos. Indeed, Kantian personhood is achieved at *some *point in the course of the physical development of a human being; the acquisition of the intelligible self, personhood, will no doubt *coincide *with (but will not be *caused *by) some natural occurrence. It is possible that some argument can be made in favor of ascribing Kantian personhood to human embryos using other aspects of Kant's work. Sagoff maintains, for example, that the question of the moral status of the human embryo could be approached from an anthropological standpoint:

In Kantian terms, a problem like that of the status of [extracorporal human embryos] belongs to the study of anthropology. According to Kant (1798/1978), "A systematic doctrine containing our knowledge of man (anthropology) can either be given from a physiological or pragmatic point of view." The physiological perspective "aims at the investigation of what nature makes of man." Nature makes nothing of man that provides him moral status or significance. To study humanity wholly in context of its potential under the aspect of the biological science – or any science – is to exclude the normative entirely [40].

Perhaps this may incite Oduncu to argue in favor of the moral status of the human embryo more from an anthropological standpoint than a biological one. Nevertheless, philosophers such as he and Novak simply assume, rather than argue, that a Kantian person begins to exist due to the empirical event of conception. I have argued that there are difficulties in making such a claim from a Kantian framework. I am not maintaining that it is impossible to make *some *argument in favor of ascribing personhood to blastocysts given a Kantian framework, only that the arguments proposed by these philosophers are fraught with difficulties that need to be, at the very least, addressed in some serious fashion. Furthermore, what is also needed is an argument as to why the beginning of the Kantian person coincides with conception, rather than some other empirical occurrence (e.g., the occurrence of brain waves in the human fetus). I now pass the buck back to these philosophers so that they can address these difficulties.

## Some Kantian considerations in favor of embryonic stem cell research

In what follows, I will present some Kantian considerations that may favor using embryos for stem cell research. It should be noted that in this section, I will give some reasons for believing that human embryos are not Kantian persons (mostly, I will borrow from Allen Wood's interpretation of Kantian philosophy). However, I am open to revising the argument in light of any possible response I may receive from philosophers that share Oduncu's contention that human embryos should indeed count as Kantian persons.

Allen Wood argues that Kant's theory is "notoriously... *logocentric*, by which I mean that it is based on the idea that rational nature, and it alone, has absolute and unconditional value" [41]. Wood takes what is usually called the "restrictive view" of Kant's moral philosophy, i.e., that Kant means to include only *actually rational *beings (beings who possess the current capability to reason) as part of the moral community. Recall Kant's definition of "person" above; only actually rational beings can be the type of beings who exercise freedom and whose actions can be imputed onto them. Only actually rational beings can possess and exercise a good will; only they can follow the moral law with duty being the sole motivator. According to the "restrictive view," when Kant argues that we must respect humanity within persons or human beings, he spoke of respecting the actual capacity to make rational choices. In this sense, Wood is right when he maintains that the Kantian notion of humanity "does not refer to membership in any particular biological species" [42]. As such, Wood would disagree with Oduncu's contention that the formula of humanity applies to all human beings simply *qua Homo sapiens*.

Kant's insistence that only actually rational or actually self-conscious beings possess moral status and inherent dignity is very much reflected in his discussions concerning the moral status of nonhuman animals. In a section in his *Lectures on Ethics *entitled "Of Duties to Animals and Spirits," Kant argues that the reason that we lack any direct duty toward animals is precisely because they lack self-consciousness:

...since all animals exist only as means, and not for their own sakes, in that they have no self-consciousness, whereas man is the end, such that I can no longer ask: Why does he exist?, as can be done with animals, it follows that we have no immediate duties to animals; our duties towards them are indirect duties to humanity [43].

All seeming duties toward animals are really indirect duties toward persons; we perform any good action towards an animal, really, for the sake of other persons, not for the animal itself.

This does not entail, however, that we are allowed to treat animals cruelly. In the same section, Kant maintains that an owner is to care for his old dog that has served him faithfully throughout many years. The reason is not because the human would be "in breach of any duty to the dog, since the latter is incapable of judgment" [44], but rather because the dog's faithful behavior toward its owner is, what Kant calls, an "analogue of merit" [45]; given that it strongly resembles the human act of merit, it should be treated and rewarded as such. The reason we should do so is not because we owe it to the dog, but rather it is because when a person is cruel or unkind toward animals, he "damages the kindly and humane qualities in himself, which he ought to exercise in virtue of his duties to mankind. Lest he extinguishes such qualities, he must already practise a similar kindliness toward animals" [46]. In other words, we should be kind to animals because doing so instills within us the virtues of kindness and humaneness, and this, in turn, will help to ensure that we will act in a kind and humane manner toward other persons. Cruel actions done onto animals are sufficiently analogous to cruel actions done onto persons (given that animals are "analogues of humanity") so that the more we practice the former, the more likely we are to practice the latter. Nevertheless, although Kant advocates the humane treatment of animals, he still maintains that we have no direct duties toward beings that lack self-consciousness and rationality, as human blastocysts do.

Of course, if Kant really does argue that only actually rational or actually self-conscious individuals are part of the moral community (and as such that the formula of humanity only applies to these individuals), this not only fares badly for nonhuman animals, and human embryos or fetuses, but also for other members of the community that we typically deem worthy of moral status, e.g., human infants, the senile, or the severely mentally disabled. That is, if Kant's theory really endorses the claim that only actually rational or self-conscious beings have moral status, we would have no more duties toward infants and the severely mentally disabled than we have toward nonhuman animals. This would not license us to treat them cruelly, as it does not license us to treat animals cruelly, but certainly the formula of humanity would not apply to them, as it does not apply to nonhuman animals. Moreover, they would not be regarded as persons with intrinsic dignity, but rather they would be regard as mere "things":

The fact that the human being can have the "I" in his representations raises him infinitely above all other living beings on earth. Because of this he is a *person*, and by virtue of the unity of consciousness through all changes that happen to him, one and the same person – i.e., through rank and dignity an entirely different being from *things*, such as irrational animals, with which one can do as one likes [47].

Needless to say that if human infants, the elderly senile, or the severely mentally disabled were regarded as mere things, to be used as instruments or as a person would wish, this would indeed be a reason to reject Kant's view of moral status. It is because of this worry that Wood argues that Kant himself misinterpreted the very spirit of his own moral theory; that, if interpreted correctly, some nonrational human beings certainly should be treated as ends in themselves even if they technically lack Kantian personhood.

Wood supports the logocentrism of Kant's moral philosophy, but argues that it need not lead Kant to the conclusion that only *actually rational *beings are ends in themselves. This consequence only arises because Kant also accepts what Wood dubs the "personification principle," which states that "rational nature is respected only by respecting humanity *in someone's person*, hence that every duty must be understood as a duty to a *person *or persons" [48]. That is, according to the personification principle, the only way of respecting rational nature is by respecting persons (human and nonhuman alike) who are actually rational. However, according to Wood, this is not the lone way of expressing such respect:

... logocentric ethics, which grounds all duties on the value of humanity or rational nature, should not be committed to the personification principle. It should hold that honoring rational nature as an end in itself sometimes requires us to behave with respect toward nonrational beings if they bear the right relations towards rational nature. Such relations... include having rational nature only potentially, or virtually, or having had it in the past, or having parts of the necessary conditions of it [49].

For example, part of respecting rational nature is respecting its potential within a young child, say an infant, even if the infant lacks self-consciousness, or the concept of an "I." As Wood notes, "it would show contempt for rational nature to be indifferent to its potentiality in children, and to treat children as mere things or as a mere means to the ends of those beings in whom rational nature is presently actual" [50]. That is, although young children are not, strictly speaking, Kantian persons, their potential rationality bears the "right relation" to actual rationality, so that they must be treated as ends in themselves, partially in order to ensure that their rational nature is cultivated and honed.

Wood draws an analogy between what he proposes here and the way in which traditional theists worship God. Such worship does not entail *only *worshipping God Himself, but also behaving in certain ways toward other beings. It is often said that by behaving beneficently toward our fellow humans, by forgiving their trespasses, and by loving one's neighbor, one is also serving God. This is because fellow human beings "stand in certain salient relations to God, such as being his creatures or being made in his image. These relations to God which make our conduct toward them expressive of our love for and devotion to God" [51]. Likewise, Wood argues, proper respect towards rational nature need not be limited to respecting only actual human persons:

Of course we should respect rational nature *in *persons, and this means respecting the persons themselves. But my main argument here depends on saying that we should *also *respect rational nature *in the abstract*, which entails respecting fragments of it or necessary conditions of it, even where these are not found in fully rational beings or persons" [52].

As a result of this contention, Wood concludes that a reasonable interpretation of Kant's notion of respect for rational nature entails that small children or individuals with mental impairments may not be treated as mere things, or mere instruments; that they also be treated as ends in themselves. These individuals should be regarded as persons in the extended sense, rather than in the actual sense. Neglecting to respect these human nonpersons as ends in themselves illustrates:

...contempt for rational nature... Kant's principle might even dictate giving priority to [the] development [of rationality] in children over promoting some of the ends of actual rational beings. It might, for example, require adults to devote scare resources to protecting, caring for and educating small children, instead of using these resources to satisfy their own contingent ends. Similar points might be made about respecting rational nature in people who have temporarily lost it through disease or injury. It would show contempt for rational nature not to care about them, and to do nothing to help them recover their rational capacities [53].

Given that Wood has eroded what he takes to be a central (but misguided) tenet of Kantian moral philosophy concerning who possesses moral status, could Wood's expansion apply to human embryos? If we are willing to broaden Kant's conception of the moral community to make room for children and individuals with mental impairtments, because these individuals also deserve to be treated as ends in themselves given their salient relationship to rational nature, can we not broaden it further to include human blastocysts? I would anticipate that philosopers like Novak and Oduncu may present such a challenge.

It is hard to tell how widely Wood means to broaden the moral community. He does not offer any necessary or sufficient conditions for when potential rationality ought to be regarded as an end in itself. Nevertheless, I doubt he means his expansion to cover human blastocysts, given that Wood does briefly criticize speciesism and maintains that it is no more morally defensible than racism:

Being a member of a certain biological species, as many animal rights advocates correctly point out, is not a sufficient reason; if we try to justify it by the fact that they are members of *our *species, then this seems no more justifiable than (and objectionable for precisely the same reasons as) giving certain people special status because they are members of our race or nationality [54].

In *Kantian Ethics*, Wood argues that "to regard all humans as persons because they are members of *our *species seems no better than regarding as persons only those who share our nationality or religion or skin color. To argue that certain entities are persons because they are members of a rational species, when they are not in fact rational beings, is no better than arguing that children are already human adults because they belong to a species whose mature members are human adults" [55]. It seems rather clear, then, that Wood does not mean to include human blastocysts in the moral community, given that they are, in essence, newly fertilized eggs that are very early in development. Indeed, at this stage they have not even developed into the embryo *proper *and the embryonic auxiliary tissue, and their individuality *qua *human beings has not yet been established (given that twinning or even chimeras can take place until approximately fourteen days post-conception). Therefore, although Wood does argue that potential rationality can bear a salient relation to actual rationality, so that beings who possess the former may still be treated with the dignity of the latter, he does not argue that embryonic potential is sufficient for fulfilling this salient relation. This does not entail, however, that the human blastocyst is not worthy of any respect at all; I intend to show below that there is a manner in which they can be respected even if they are indeed used for research purposes.

The reason Wood argues this is because a human embryo or fetus possesses one trait that infants and children do not in terms of their potential rationality: an embryo or (a pre-viable) fetus cannot develop into an infant without the use of a specific person's womb. Thus, to bestow upon them the extended status of full personhood, even though they are not technically persons in the Kantian sense, may entail that some *actual *persons, i.e., women who have made the rational, autonomous, decision not to gestate, would be instrumentalized and treated as mere means rather than as an ends in themselves. If we are to respect the rational nature within Kantian persons, this entails that "rational beings should not be subjected to deception *or coercion*" [56]. According to Wood, if bestowing extended personhood onto beings that are not technically persons results in violating the dignity of actual persons, then proper respect for rational nature does not necessitate that we grant that extended personhood. Wood writes:

... consider the question of whether a fetus, like an infant, is to be regarded as a person in the extended sense. The question should turn not only on whether our conduct duly respects the value of the (still merely potential) personhood (in the strict sense) of the fetus, but also on whether it duly respects the dignity of actual persons in the strict sense – in particular, the dignity of the person in whose body the fetus is developing. If that person is forced to bear a child she does not want, or if her right to control the life process going on in her body is coercively restricted by others... then their conduct expresses extreme disrespect for the right of rational nature in her person [57].

There are currently approximately half a million frozen embryos leftover in fertility clinics across the United States of America. To regard those embryos as persons would mean that as much as possible must be done to bring those embryos into fruition, especially since prolonged exposure to freezing temperatures may compromise their viability and potential, and therefore would effectively terminate them. This may entail implanting those embryos into the uteruses of women, whether or not they desire to gestate the embryos (at least until ectogenesis becomes a reality, in which case the embryos could be gestated without the use of a woman's womb). As Wood rightly points out, however, such an action would disrespect the actual capacity for humanity that women possess. If "granting to embryos and fetuses the same "right to life" that is thought to belong to persons in the extended sense would involve such coercive or invasive conduct, then it would constitute a gross disrespect to rational nature to grant them that status" [58].

I believe that Wood's overall argument is correct, but the question remains, then, how *should *we treat human embryos from a Kantian perspective? What would a Kantian argument in favor of embryonic stem cell research look like? In order to adequately address this issue, I think we should adhere to Wood's standards for answering questions of this sort:

[it] depends on how far our conduct... expresses due respect for the dignity of rational nature, and how far it falls short of this... [i]t is relevant to the right answer to such questions not only how we are acting toward rational nature in our treatment of human beings who are not persons in the strict sense, but also whether in our conduct we duly respect this value in those who are persons in the strict sense [59].

Even though human embryos are potential persons to some extent, once they are slated for destruction at fertility clinics, their potential has effectively vanished.^g ^As Ted Peters and Gaymon Bennett write, embryos that reside in a Petri dish or in a frozen state possess some of the necessary traits that would enable them to grow into infants, but they lack one very important one: they do not inhabit the proper environment that would facilitate their growth into persons:

As many supporters of embryonic stem cell research have rightly pointed out, the argument from potentiality assesses the status of the embryo in accordance with the presumption that the embryo can and will be placed in vivo. The potential for an embryo in the lab to become a baby is nil. This is not a criticism, moral concession, or an argument from geography (as critics rhetorically put it). It is an ethically relevant fact. An embryo in vitro has many intrinsic qualities that are needed for baby making, but it does not have all of the necessary qualities. Although it has DNA, at minimum it still needs a womb to proceed down the developmental pathway [60].

Therefore, the following is the appropriate manner of looking at the embryonic stem cell research debate through the lens of a Kantian framework: If we must decide between discarding surplus embryos in fertility clinics (and by "surplus," I mean that the embryos have no possibility of being implanted into a womb; their genetic parents do not wish to implant them themselves and they refuse to give permission to allow the embryos to be adopted) or using them for very promising research, which of these two options most "expresses due respect for rational nature?"

The same fate that befalls nonhuman animals seems to befall human embryos in this regard. Given that they are not currently self-conscious nor rational beings (indeed, they have not even developed the necessary neural apparatus to have any thought or sentience whatsoever), because their potential to develop into persons has effectively vanished once they are slated for destruction, and because granting them extended personhood would entail a gross disrespect of actual personhood, they can be used as means to the ends of persons. But, does this mean that we can use them in *any *which way we choose? That does not follow from Kant's philosophy any more than the wanton treatment of animals follows.

John Robertson writes that in vitro embryos are symbolically very powerful. Even those embryos that are slated for destruction and "will not be placed in the uterus have some meaning in this regard, for they operate as a symbol of human life or constitute an arena for expressing one's commitment to human life" [61]. In Kantian terms, embryos can be said to be "analogues" to human persons, and certainly there is "some value in the potential personhood of an embryo or fetus" [62]. After all, Kant may have argued, our empirical, phenomenal, self did begin at conception (or shortly thereafter, if one believes that irreversible individuality is necessary for the existence of the empirical self. If one does believe this, the empirical self may not come into existence until the twinning/chimera stage has passed). Because they are such analogues, they do seem to deserve some esteem in this regard, if only to find a way of respecting their symbolic value.

Such respect is certainly not being manifested with our current treatment of embryos at fertility clinics. It is difficult to see how incinerating them or flushing them down a drain at fertility clinics expresses respect for rational nature, especially since the outcome to doing this may be to compromise the advancement of a kind of research that can indeed benefit so many rational beings. Moreover, it is inconsistent to invoke the formula of humanity as an argument against the moral permissibility of embryonic stem cell research but not against the moral permissibility of current In Vitro Fertilization (IVF) techniques in the United States of America, which deliberately produce more embryos than will be transplanted into a uterus for possible implantation. Embryos are being regarded solely as a means to an end here; they are created and then used for no other reason other than to produce infants for otherwise infertile couples. In the very same speech where Bush argues that we should not treat embryos as mere means for our scientific ends, he lauds IVF practices for its role in relieving infertility. To truly treat embryos as ends in themselves would require a cessation of current IVF techniques in the United States of America so that more embryos are not overproduced and later destroyed. Until we are willing to do this, however, we cannot use Kant's formula of humanity as a definitive argument against the moral permissibility of embryonic stem cell research if we are unwilling to apply the same criticism against current IVF techniques. Surely if we are willing to instrumentalize embryos in order to relieve infertility, which is classified as a disease, we should be willing to instrumentalize them to help in the fight of alleviating other debilitating diseases. It is not at all clear to me that embryos may be instrumentalized in order to treat the disease of infertility, but that they cannot be instrumentalized in order to treat Alzheimer's disease, Parkinson's disease, spinal cord injuries, retinal deterioration, or diabetes. Certainly alleviating the former disease is a worthy cause, but not much more than alleviating the latter five (amongst the many other afflictions that embryonic stem cell research has the potential to treat).

Elsewhere, I have argued that using embryos for stem cell research, like using anencephalic infants as organ donors, is far more respectful than letting their lives go to waste at the bottom of drains in IVF clinics. If we want to treat embryos as analogues to persons, we should try to treat them in a way that, at least, cultivates our character so that we can better regard our fellow persons, as Kant said we are to do with nonhuman animals. Flushing embryos down a drain does not at all cultivate such a character, but using them for stem cell research may.

Using human embryos for research will allow scientists, and us as a society, to engage in and endorse a practice that has as its intent the hope of curing many painful diseases, for example, Alzheimer's disease, which progressively eradicates our rational faculties. Certainly it respects rational nature to strive to find cures for certain diseases that destroys rational nature; it does not seem far-fetched to argue that, from a Kantian perspective, respecting humanity or rational nature would entail attempting to preserve it in the face of disease. Indeed, recall Wood's point that it would be disrespectful to the disintegrating rational nature in persons afflicted with disease to do "nothing to help them recover their rational capacities." It seems to me that discarding surplus embryos at fertility clinics, rather than using them for research that may aid in the cure of a disease like Alzhiemer's, seems to run dangerously close to doing just that.

Moreover, by alleviating human suffering and disease, we are fulfilling our duty of beneficence toward others (a duty that Kant acknowledges we all possess), and we are, in a sense, taking on the ends of the sick and diseased as part of our own ends (for I am sure that part of their ends is to find relief from, and cures for, their afflictions). Nothing of this can be achieved if we continue to wantonly create and discard embryos for use in fertility treatments, and certainly we are not treating embryos in any respectful manner or regarding them as Kantian persons by doing so. As long as we are willing to instrumentalize embryos for IVF purposes, consistency demands, from a Kantian perspective at least, to use them for stem cell research in order to further the health and improve the lives of actual persons.

## Endnotes

a) Many thanks to Dr. Allen Wood for pointing this out via e-mail correspondence.

b) There can be controversy here regarding what Kant means by the term "capacity" or "predisposition." If Kant means to use the term "capacity" in order to denote current capabilities, so that a typical adult human has the capacity for reason given that he can do so at the current time if he so chooses, then obviously human fetuses and embryos do not have the capacity for reason, given that no fetus nor embryo possesses the current capability to reason. Allen Wood, for example, perhaps would interpret Kant's use of the term in this way, given that, as we will see later in the paper, Wood does make a distinction between beings who actually possess the current capability to exercise rationality and beings that have the potential to be rational (such as fetuses and neonates); he refers to the former as beings who possess rationality in the "technical" or "strict" sense and the latter as beings who possess rationality in the "nontechnical" or "extended" sense. Other philosophers, however, may interpret Kant's use of the term "capacity" to mean "potential," so that the human embryo or fetus does possess the capacity or the predisposition for reason because it possesses this potential given that it consists of a healthy human genetic code. In this paper, given that I side with Wood on various points, I will also side with him concerning Kant's use of the term "capacity," although I recognize that this is a reading that is very much open to debate; to enter this debate, however, would take me beyond the scope of my objective here.

c) The distinction between humanity and personality can be interpreted as being more of a difference in emphasis than in kind. Although the predisposition for humanity is defined as the capacity for reason *proper *and the predisposition for personality is defined as the capacity to follow the moral law, the capacity for reason *proper *entails the capacity for autonomy as a necessary precondition, which, in turn, entails the capacity to engage in moral actions. Therefore, the two ought not to be looked upon as completely distinct capacities or predispositions, rather their differences can be viewed as focusing on different aspects of our general capacity for reason and to be autonomous beings. I would like to thank to one of my anonymous reviewers for pointing this out to me.

d) This method of argumentation would assume, therefore, that Kant means to use the terms "capacity" or "predisposition" in order to denote the *potential *to reason, rather than to denote the *current capability *to reason. Although I am siding with Wood's interpretation of "capacity" and "predisposition" here, I will illustrate in what follows that there are difficulties in supporting the view that Kant would maintain that the capacity or predisposition for humanity is present from the moment of conception, regardless of how one chooses to define these terms. More precisely, because the capacity for reason is intimately connected with transcendental freedom, the capacity for reason, from a Kantian framework, is a supersensible capacity, and it is difficult to illustrate, using Kant's philosophy, that a supersensible capacity can be instantiated in the world due to a natural event, e.g., conception.

e) See Patrick Kain, 'Kant's Defense of Human Moral Status'. Kain's view seems to heavily depend on Kant having a certain teleological conception of human nature, i.e., that all human beings, from the origin of their organisms, are ensouled with the nature that is common to their species. This would mean that the embryo has the capacities and predispositions of humanity and personality from the genesis of the human organism, which can be denoted either at conception or irreversible individuality, which takes place at approximately fourteens days post-conception. If the latter is when one takes the human organism to be formed (when twinning is no longer possible and cleaving has ceased), then this poses no obstacles for the moral permissibility of human embryonic stem cell research, given that in vitro blastocysts are five-days-old and will never reach the stage of irreversible individuality. Nevertheless, Kain's argument seems to conflict with other philosophers who argue that Kant did *not *have any teleological view of human nature; that he did not believe that a human being possesses "an essence which defines his true end".

f) This dualism is not to be interpreted as a type of substance or Cartesian dualism. While there is reason to believe that Kant argues in favor of the existence of an immaterial soul that makes up our essential identity, we cannot derive this thesis from the discussion here alone.

g) Such a view is akin to the Jewish conception of the moral status of extracorporeal embryos. For example, in his testimony to the National Bioethics Advisory Commission, Rabbi Elliot Dorff, Ph.D. argues that extracorporeal embryos have no legal or moral status outside the womb under Jewish law because " [o]utside of the womb... they have no such potential" to become persons.

## Competing interests

The author(s) declare that they have no competing interests.
